# Medical News

**Published:** 1878-07

**Authors:** 


### Medical News.

---

THE *Metric System of Weights and Measures* is now officially introduced into the United States Marine Hospital service. As it is quite likely to be adopted, sooner or later, by the profession throughout the country, the following very simple rules and suggestions contained in circular No. 3, series 1878, of the Superintendent Surgeon-General, U. S. M. H. S., for the ready conversion of the present weights and measures into their respective equivalent in metric terms may be of some interest to our readers.

RULES FOR CONVERTING TERMS OF THE UNITED STATES APOTHECARIES' WEIGHTS AND MEASURES INTO THEIR RESPECTIVE EQUIVALENTS IN TERMS OF THE METRIC SYSTEM.

1.—To express quantities by weight of the Apothecaries' system in metric terms, or to write medical prescriptions in metric weights.

*Rule A.—Reduce each quantity to grains ; then divide the number by* 10 (or move the decimal point one place to the left), *and from the quotient subtract one-third.* The remainder is in each case the number of grammes representing (nearly) the same quantity. Or,

*Rule B.—Reduce each quantity to drachms, and multiply the number by* 4. The product is in each case the number of grammes representing (nearly) the same quantity. Or,

*Rule C.—Reduce each quantity to ounces, and multiply the number by* 32. The product is in each case the number of grammes representing (nearly) the same quantity.

2.—To express quantities by measure of the Apothecaries' system in metric terms, or to write medical prescriptions in metric cubic measures.

*Rule D.—Reduce each quantity to minims, then divide the number by* 10 (or move the decimal point one place to the left),

*and from the quotient subtract one-third.* The remainder is in each case the number of cubic centimeters representing (nearly) the same quantity. Or,

*Rule E.—Reduce each quantity to fluid drachms, and multiply the number by* 4. The product is in each case the number of cubic centimeters representing (nearly) the same quantity. Or,

*Rule F.—Reduce each quantity to fluid ounces, and multiply the number by* 32. The product is in each case the number of cubic centimeters representing (nearly) the same quantity.

The following prescription—

℞: Potassii Bromidi, ℥i.

Elix. Aurantii, fl. ℥viij.

Mix—

would, in metric terms, be written :

℞: Potassii Bromidi, 32 Gm.
Elix. Aurantii, 250 C. C.
Mix.

Or, in more finished decimal manner:

℞: Potassii Bromidi, 30 Gm.
Elix. Aurantii, 250 C. C.
Mix.

The exact equivalents of the grain, drachm, and ounce (troy), in grammes; of the gramme in grains; of the minim, fluid drachm, and fluid ounce in cubic centimeters, and of the cubic centimeter in minims are as follows:

1 grain, troy, is equal to 0.065— grammes.
1 drachm, troy, is equal to 3.888— grammes.
1 ounce, troy, is equal to 31.103 + grammes.
1 gramme is equal to 15.43234874 grains, troy.—*Prof. Miller.*
(1 avoirdupois pound is equal to 453.592 + grammes.)
(1 avoirdupois ounce is equal to 28.350 + grammes.)
1 minim is equal to 0.062— cubic centimeters.
1 fluid drachm is equal to 3.697— cubic centimeters.
1 fluid ounce is equal to 29.573— cubic centimeters.

In preparing prescriptions the following approximate equivalents may be substituted: 1 grain, 0.06 Gm.; 1 drachm, 4 Gm.; 1 ounce, 30 Gm.; 1 minim, 0.06 C. C.; 1 fluid drachm, 4 C. C.; 1 fluid ounce, 30 C. C.—

